# Retrospective cohort study of rough-surface titanium implants with at least 25 years’ function

**DOI:** 10.1186/s40729-017-0101-7

**Published:** 2017-09-05

**Authors:** Tadashi Horikawa, Tetsurou Odatsu, Takatoshi Itoh, Yoshiki Soejima, Hutoshi Morinaga, Naruyoshi Abe, Naoyuki Tsuchiya, Toshikazu Iijima, Takashi Sawase

**Affiliations:** 1 Kyushu Implant Research Group, 4-14 Kokaihonmachi, Chuo-ku, Kumamoto, 860-0851 Japan; 2 Department of Applied Prosthodontics, Graduate School of Biomedical Sciences, Nagasaki University, 1-7-1 Sakamoto, Nagasaki, 852-8588 Japan

**Keywords:** Dental implants, Implant-supported prosthesis, Long-term survival, Titanium plasma-sprayed surface

## Abstract

**Background:**

The longitudinal clinical outcomes over decades contribute to know potential factors leading to implant failure or complications and help in the decision of treatment alternatives.

**Methods:**

The cases of all patients who received dental implants treated with titanium plasma-sprayed surfaces and whose prostheses were set in the period 1984–1990 at seven private practices were retrospectively analyzed. The cumulative survival rate, the cumulative incidence of peri-implantitis, and the complication-free prosthesis rate were calculated with Kaplan-Meier survival curves, and the factors’ influence on implant survival rate and the incidence of peri-implantitis were determined by a single factor in univariate analyses and multivariate analyses.

**Results:**

A total of 223 implants and 106 prostheses were applied to 92 patients, and approx. 62% of the implants and patients dropped out over the 25 years following their treatment. The cumulative survival rates of the implants at 10, 15, and 25 years were 97.4, 95.4, and 89.8%, respectively. A significant difference was observed in the implant position. The cumulative incidences of peri-implantitis at 10, 15, and 25 years were 15.3, 21.0, and 27.9%, respectively. Significant differences were observed in the gender, implant type, and width of keratinized mucosa around the implant. The cumulative survival rates of mechanical complication-free prostheses at 10, 15, and 25 years were 74.9, 68.8, and 56.4%, respectively. The difference in the type of prosthesis resulted in significant differences.

**Conclusions:**

The high rate of dropout during follow-up indicates the difficulty of determining long-term (> 25 years) prognoses. The gender, location, and width of keratinized mucosa affected the development of peri-implantitis, resulting in late failures. Implant-supported overdentures were frequently repaired. Tooth implant-supported prostheses are not recommended for long-term survival.

## Background

Dental implant treatment based on the concept of osseointegration [1] is now a widely accepted restorative treatment for fully and partially edentulous patients. In the earliest days of the use of osseointegrated implants, two different topographies were applied on the implant surfaces: a machined minimally rough titanium surface such as the Brånemark system and a rough microporous titanium plasma-sprayed surface such as the ITI system [2]. In clinical studies, the long-term (i.e., up to 20 years) survival rate of Brånemark-system implants was in the range of 80–99% [3–5] and that with ITI-system implants was 88–96% [6, 7].

Despite the high survival rates, implant-supported restorations are still subject to biological and mechanical complications. The focus in dental implant treatments has shifted from implant survival to (1) implant success, (2) peri-implant infection, and (3) long-term outcomes of prostheses. Since the increasing human life expectancy and most of the patients who undergo implant treatment are middle-aged (approx. 40–60 years old) [8, 9], the determination of these longitudinal clinical outcomes over decades will contribute to the evaluation of treatment alternatives.

The aim of this retrospective study was not only to evaluate the long-term outcomes of solid-screw implants with a titanium plasma-sprayed (TPS) surface but also to assess the survival rates associated with the biological and mechanical complications.

## Methods

### Study design

This retrospective observational study was approved by the ethical committee of Nagasaki University (No. 1512). The cases of all of the patients who underwent dental implant treatment with a TPS-surfaced solid-screw implant and whose prosthesis was set in the years 1984–1990 at seven private practices were analyzed. All inserted implants were either a TPS-type (TPS-type, Institute Straumann, Basel, Switzerland) implant or a BONEFIT 45° shoulder-type (S-type, Institute Straumann) implant. We identified a total of 223 implants inserted into 92 patients.

### Medical record assessment

Medical records were reviewed, and the patient-related parameters of age, gender, smoking habit, the date of implant surgery, and the date of the prosthesis setting were collected. The information of implant (length, diameter, type), site of implantation, width of keratinized mucosa, and additional pre- and/or post-implant surgery (i.e., bone augmentation, soft tissue management) was also collected. The types of prostheses were classified into implant-supported fixed prostheses, tooth implant-supported fixed prostheses, and implant-supported overdentures.

The endpoint of this study was set at December 31, 2015. Episodes of implant failure, biological complication (i.e., peri-implantitis with suppuration), and mechanical complications (i.e., component or laboratory-fabricated suprastructure’s failure) were recorded.

### Statistical analysis

JMP Pro software (ver. 11.2.0, SAS, Cary, NC, USA) was used for the statistical data analyses. The cumulative implant survival rate, the cumulative incidence of peri-implantitis, and the cumulative “complication-free” survival rate of implant-supported restorations were analyzed using the Kaplan-Meier survival estimator method. The cumulative “mechanical complication-free” survival rate of implant-supported restorations was estimated by a restoration-based analysis. The influence of the following variables on the implant survival rate and the incidence of peri-implantitis were determined by a single factor in univariate analyses (Kaplan-Meier) and multivariate analyses (Cox proportional hazards regression analysis): patient gender, smoking habit, implant type (S-type or TPS-type), implant position (three categories: maxilla, anterior mandible, posterior mandible), presence of additional soft tissue management (i.e., free-gingival graft, vestibular extension, and frenectomy), and the width of keratinized mucosa around implant (> 2 mm). The influence of patient gender and type of prosthesis on the complication-free survival rate of implant-supported restorations was determined. The results were considered statistically significant at *p* < 0.05.

## Results

### Patient cohort

**Fig. 1:**
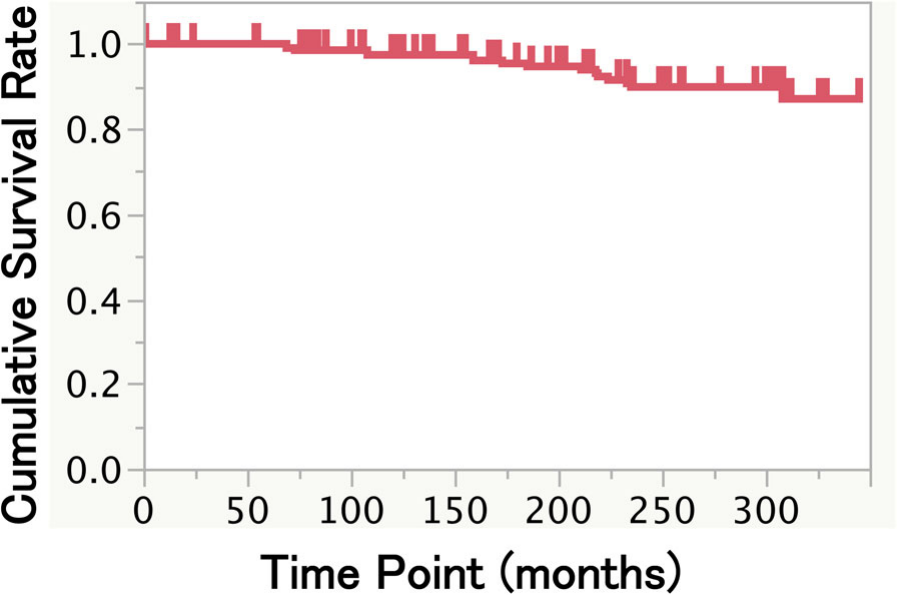
Kaplan-Meier cumulative survival rate at 10, 15, and 25 years after the prosthesis setting

A total of 92 patients (38 men, 54 women; mean age 54.3 years, range 20–78) received implant-supported prostheses (at the seven private practices) between 1984 and 1990. The distribution of patients by age and gender is presented in Table 1. Fifty-seven patients (140 implants) were considered dropouts due to the fact that no data were obtained at the endpoint, but 25 years had passed since the prosthetic treatment delivery of one dropout patient (with four implants). The Kaplan-Meier estimate shows the censorings (Fig. 1), and they present unbiased throughout the observation period. The dropout reasons for 42 patients were illness, moved away, and not showing up for check-ups; another 15 patients had passed away before the present analysis.

**Table 1:** Age and gender distributions (*n* = 92)

Age/gender	Male	Female	Total
20–29	3	1	4
30–39	2	7	9
40–49	8	14	22
50–59	8	18	26
60–69	15	13	28
70–79	2	1	3
Total	38	54	92

### Implant diameter, length, and location

A total of 223 implants were placed in 15 fully edentulous patients and 77 partially edentulous patients. The distributions of implants by diameter, length, and location are presented in Tables 2, 3, and 4. Twenty-four implants were placed in the maxilla (10.8%), and 199 implants were placed in the mandible (89.2%). Only two implants were applied to the maxillary anterior region, whereas 152 implants (68.2%) were applied to the mandibular posterior region (Tables 2 and 3). Regarding the sizes of the implants, 4.1-mm dia. and 10-mm length were the most frequently used implant dimensions (70.9 and 39%, respectively).

**Table 2:** Distribution of implants in situ (*n* = 223)

Position	1	2	3	4	5	6	7	Total
Maxilla	0	0	2	6	6	8	2	24
Mandible	7	32	8	30	20	59	43	199

**Table 3:** Distribution of implants by diameter and location (*n* = 223)

Dia. (mm)	Maxilla anterior	Maxilla posterior	Mandible anterior	Mandible posterior	Total
3.5	1	2	15	14	32
4.0	0	0	13	17	30
4.1	1	20	19	118	158
4.8	0	0	0	3	3
Total	2	22	47	152	223

**Table 4:** Distribution of implants by length and location (*n* = 223)

Dia. (mm)	Maxilla anterior	Maxilla posterior	Mandible anterior	Mandible posterior	Total
8	0	8	3	8	19
10	0	5	9	73	87
11	1	0	1	6	8
12	1	9	7	44	61
14	0	0	13	13	26
17	0	0	11	7	18
20	0	0	3	1	4
Total	2	22	47	152	223

### Additional surgery

Four implants of one patient were inserted into the re-constructed mandible with iliac bone, due to an ameloblastoma. Additional soft tissue managements were applied to 96 implants. Free gingival graft was used for 86 implants, and frenectomy and vestibular extension were applied to 15 and 13 implants, respectively.

### Cumulative survival rate and biological complications

**Fig. 2:**
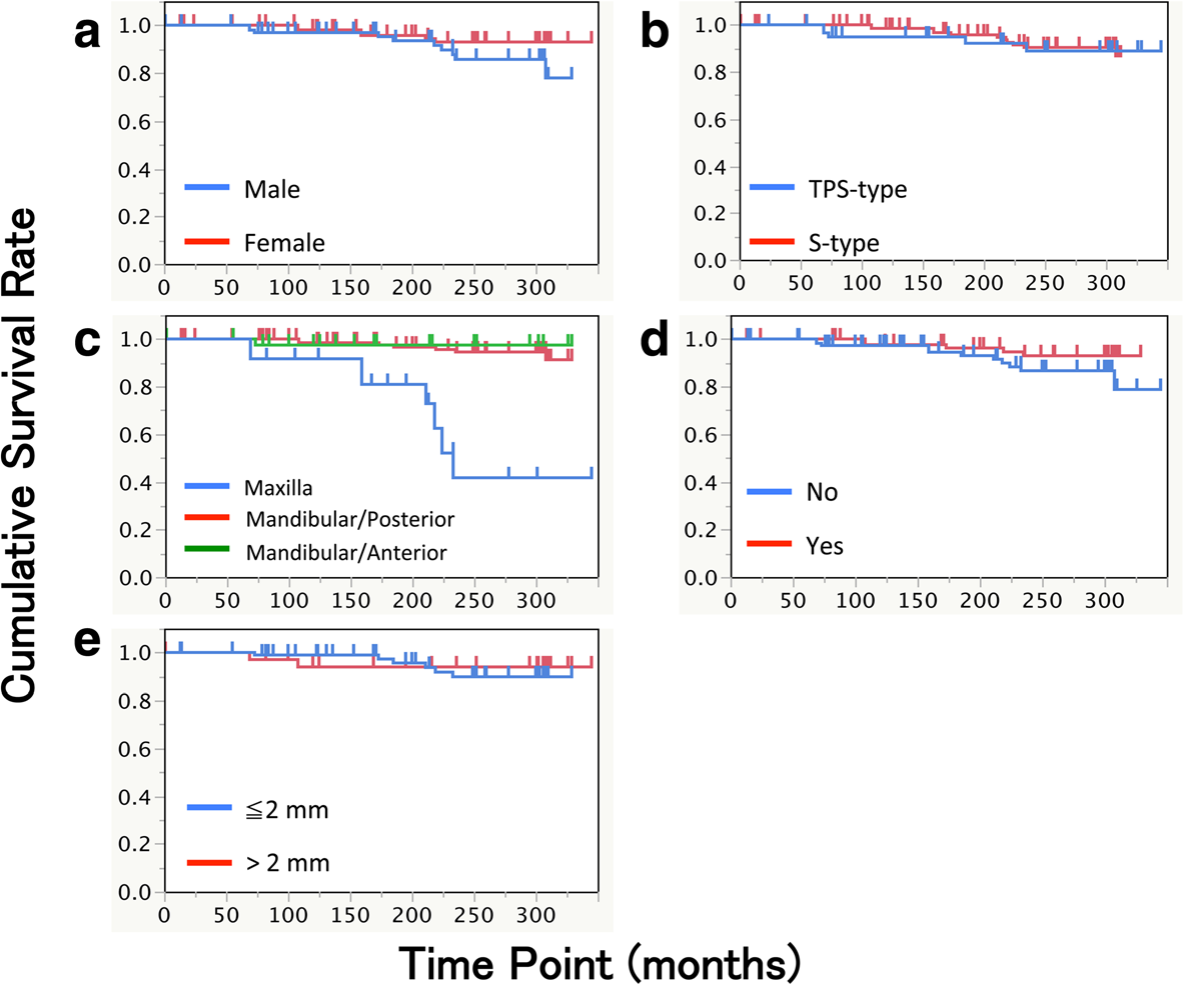
Kaplan-Meier cumulative survival rates by **a** gender (*p* = 0.1049), **b** implant type (*p* = 0.6259), **c** implant position (*p* < 0.0001), **d** presence of additional soft tissue management (*p* = 0.1149), and **e** width of keratinized mucosa around implant (*p* = 0.7132). Log rank test was used for assessing statistical significance

Sixteen implants were lost during the observation period. The Kaplan-Meier cumulative survival rates were 97.4, 95.4, and 89.8% at 10, 15, and 25 years after the prosthesis setting, respectively (Fig. 1). After stepwise backward selection, implant position in the mandibular vs. the maxilla showed the significant difference in the cumulative survival rate (Table 5, Fig. 2c). The gender, implant type, additional soft tissue management, and width of keratinized mucosa did not provide significant differences with respect to the survival of the evaluated implants in this study (Fig. 2a, b, d, and e). The reasons for late failure were peri-implant infection (14 implants) and unknown (two implants).

**Table 5:** Cox regression analyses for implant survival

	Hazard ratio	95% confidence interval	*p* value
Gender (male)	1.99	0.538~8.201	0.3018
Implant type (TPS)	2.86	0.579~13.626	0.1897
Implant position (maxilla to mandibular/anterior)	40.09	4.062~994.751	0.0012
Implant position (maxilla to mandibular/posterior)	18.69	3.127~155.409	0.0013
Implant position (mandibular/anterior to mandibular/posterior)	0.47	0.022~3.912	0.5024
Additional soft tissue management (yes)	1.64	0.290~12.695	0.5808
Width of keratinized mucosa (> 2 mm)	0.78	0.166~3.294	0.7365

**Fig. 3:**
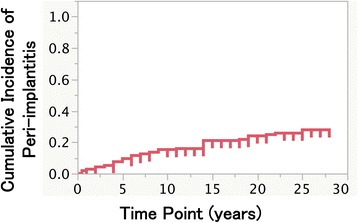
Cumulative incidence of peri-implantitis

**Fig. 4:**
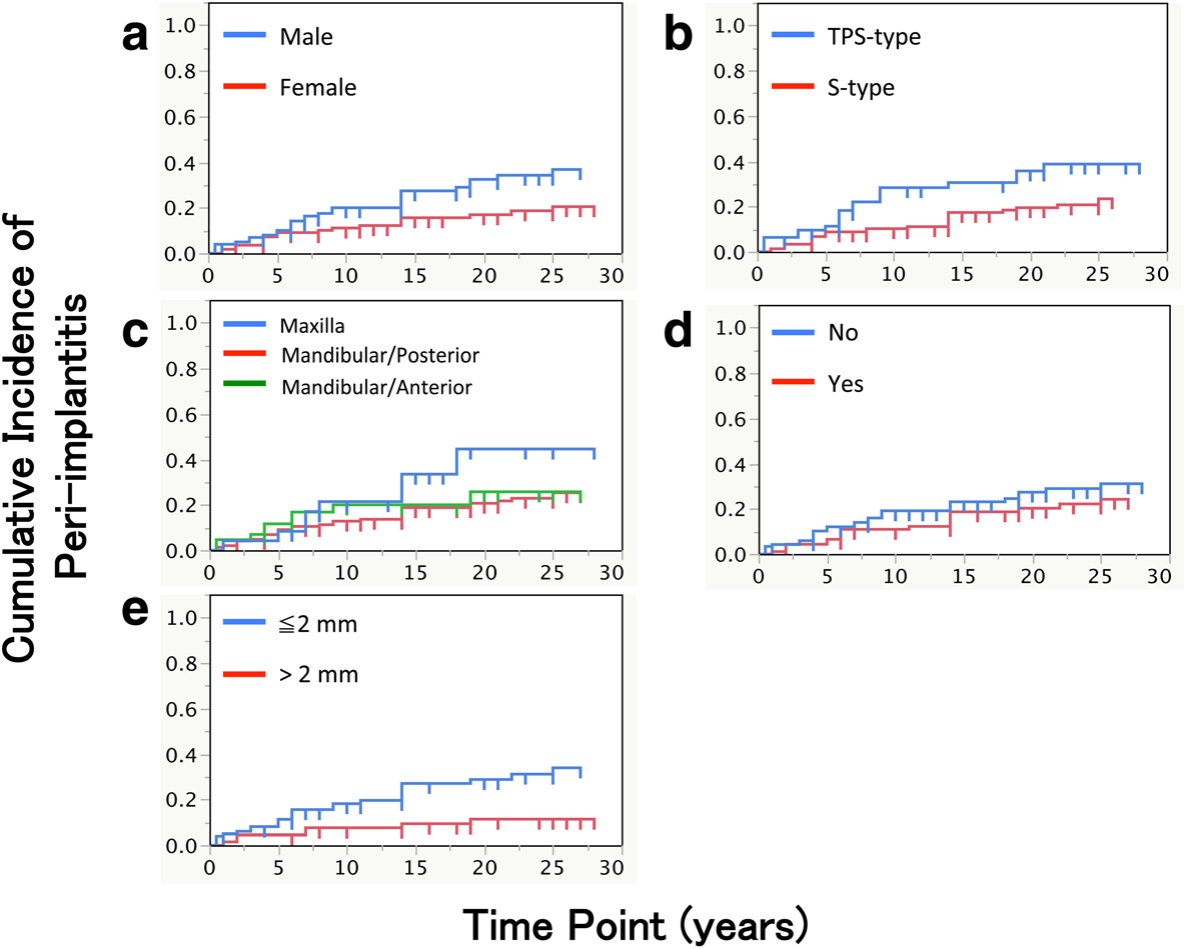
Cumulative incidence of peri-implantitis by **a** gender (*p* = 0.0221), **b** implant type (*p* = 0.0128), **c** implant position (*p* = 0.2470), **d** presence of additional soft tissue management (*p* = 0.2488), and **e** width of keratinized mucosa around implant (*p* = 0.0045). Log rank test was used for assessing statistical significance

A total of 48 implants were eventually accompanied by a peri-implant infection: the cumulative incidence of peri-implantitis was 9.5, 15.3, 21.0, and 27.9% at 5, 10, 15, and 25 years after the prosthesis delivery, respectively (Fig. 3). After stepwise backward selection, the gender, implant type, and width of keratinized mucosa showed the significant difference in the cumulative survival rate (Table 6, Fig. 4a, b, and e). The difference in implant position and additional soft tissue management did not result in significant differences with respect to the cumulative incidence of peri-implantitis (Fig. 4c, d).

**Table 6:** Cox regression analyses for cumulative incidence of peri-implantitis

	Hazard ratio	95% confidence interval	*p* value
Gender (male)	2.38	1.138~5.362	0.0208
Implant type (TPS)	4.35	1.897~9.941	0.0006
Implant position (maxilla to mandibular/anterior)	6.08	1.384~24.436	0.0188
Implant position (maxilla to mandibular/posterior)	3.45	0.903~11.111	0.0679
Implant position (mandibular/anterior to mandibular/posterior)	0.57	0.207~1.442	0.2370
Additional soft tissue management (yes)	1.18	0.535~2.714	0.6826
Width of keratinized mucosa (> 2 mm)	0.24	0.094~0.559	0.0006

### Cumulative survival rate of mechanical complication-free prostheses

A total of 106 prostheses were applied to 92 patients. Nine prostheses were single crowns, 17 prostheses were implant-supported overdentures, and the other 80 prostheses were multiunit fixed partial dentures. Thirty-seven of the multiunit fixed partial dentures were splinted with natural teeth as an abutment (i.e., tooth implant-supported fixed prostheses). With respect to the materials of the occlusal surface, 21 of the fixed prostheses were veneered with porcelain, and the other 68 were made from dental alloys (Au-Pt or Au-Ag-Pd alloys).

**Fig. 5:**
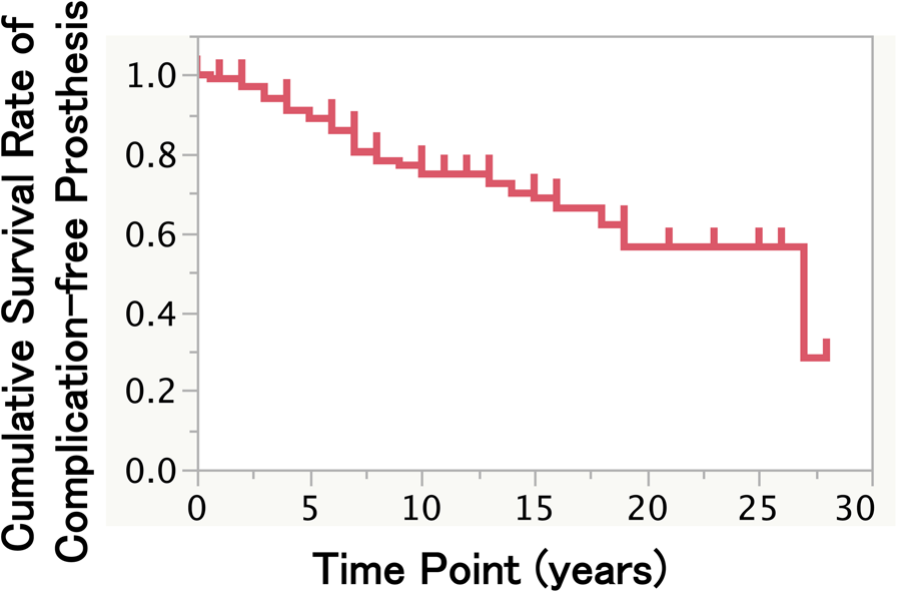
Kaplan-Meier cumulative survival rate of complication-free prostheses at 10, 15, and 25 years after the prosthesis setting

**Fig. 6:**
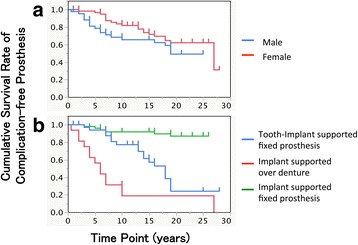
Cumulative survival rate of complication-free prostheses by **a** gender (*p* = 0.1220) and **b** type of prostheses (*p* < 0.0001). Log rank test was used for assessing statistical significance

The Kaplan-Meier cumulative survival rate of mechanical complication-free prostheses was 74.9, 68.8, and 56.4% at 10, 15, and 25 years (Fig. 5). The gender difference did not result in a significant difference with respect to the rate of mechanical complication-free prosthesis, but the difference in the type of prosthesis did (Table 7, Fig. 6a, b). For 11 of the 37 tooth implant-supported prostheses, the abutment teeth were extracted due to caries, periodontitis, or root fracture during the observation period. Regarding the implant-supported overdentures, the following mechanical complications were observed: the total number of relinings was 22 times; that of artificial tooth replacement was 17; attachment replacements were performed 15 times; bar fractures were observed in three cases, and screw loosening occurred twice.

**Table 7:** Cox regression analyses for cumulative survival rate of complication-free prostheses

	Hazard ratio	95% confidence interval	*p* value
Gender (male)	1.82	0.946~3.487	0.0725
Type of prostheses (implant-supported fixed prostheses to implant-supported overdenture)	0.04	0.013~0.108	< .0001
Type of prostheses (implant-supported fixed prostheses to tooth implant-supported fixed prostheses)	0.13	0.047~0.316	< .0001
Type of prostheses (tooth implant-supported fixed prostheses to implant-supported over denture)	0.31	0.148~0.654	0.0026

## Discussion

Although all implants used in this study were withdrawn from the market about 20 years before, the longitudinal clinical outcomes over decades will help to better understand potential factors leading to implant failure or complications and assess the safe and predictable use of dental implant. Our analyses revealed a 25-year cumulative survival rate of 89.8% after the prosthesis setting, which seems comparable to the result of a recent study [7]. Although approx. 62% of the patients and implants in our original cohort dropped out during the follow-up period, according to Table 1, a majority of patients who underwent implant treatment were middle- and old-aged (82.6% of the patients were 40–69 years old) and thus some of the patients could not continue maintenance for varying reasons over a 25-year follow-up. The other 38% of the patients are healthy and likely to visit their dentists for maintenance, and they were included in the 25-year cumulative survival rate [7]. Therefore, the true long-term survival rate might have been lower than we reported herein due to bias from the patients who dropped out.

In addition, only four implants of one patient were inserted into a re-constructed site from the iliac bone; no other bone augmentation procedure such as bone graft, guided bone regeneration (GBR), and sinus floor elevation were conducted. The principle of guided tissue regeneration (GTR) was introduced in 1982 [10], and GBR was introduced in 1988 [11]. The technique of sinus floor elevation was initially introduced in 1980 [12]. These complex augmentation procedures had not been common at that time [2], especially in private practices in Japan, and thus, they were not used for any of the patients in the present study. There is thus some degree of bias regarding the numbers of implant and the lengths of the implants according to the implant position. The number of implants applied to the maxilla anterior region was only two, since getting the esthetic result with implant prostheses was uncertain in those days. And the number of implants under 10 mm long was greater at posterior sites compared to anterior sites due to the sinus and inferior alveolar nerve.

Peri-implantitis is the major reason for late failure [13, 14]. The consensus report of the Sixth European Workshop on Periodontology described peri-implant mucositis in approx. 80% of subjects restored with implant, and peri-implantitis in 28–56% of subjects [15]. In the present study, the cumulative incidence of peri-implantitis was 9.5, 15.3, 21.0, and 27.9% at 5, 10, 15, and 25 years after the prosthesis setting, respectively. Derks and Tomasi reported a positive relationship between the incidence of peri-implantitis and the mean function time by performing a meta-regression analysis of a systematic review [16], whereas the current cumulative result shown in Fig. 3 may represent the time course of the peri-implantitis incidence. Interestingly, the incidence of the peri-implantitis increased gradually with time; the rate of increase was approx. 1–1.5% per year.

Many potential factors associated with the incidence of peri-implantitis were reported [17, 18]. In the present study, the gender, implant type, and width of keratinized mucosa were identified as risk factors. Regarding gender, Koldsland et al. also reported a male population with overt peri-implantitis [19], whereas Attard and Zarb reported that women experienced more peri-implant bone loss than men [20]. Other studies and reviews reported that gender had no effect on peri-implantitis [21, 22]. Some other gender-related factors might affect the results.

Regarding implant type, a difference between the S-types and the TPS-types is whether the existence of an abutment connection or not. The TPS-types are one-piece implants, and the S-types are two-piece but one-stage implants. Duda et al. reported that one-piece implants showed more marginal bone loss than two-piece implants [23]. In addition, a TPS surface is classified as “rough” surface when the surface roughness is more than 2 μm (Sa > 2 μm) [24]. Teughels et al. reported that a transmucosal implant surface with higher surface roughness facilitates biofilm formation [25] and thus TPS-type implants showed a higher incidence of peri-implantitis compared to the S-type.

Regarding the width of keratinized mucosa, many studies and a review have indicated that the presence of a sufficient width of keratinized mucosa is necessary for maintaining healthy peri-implants [26–29]. In the present study, when 2 mm of keratinized mucosa was used as the adequate width, the *p* value was 0.053 (data not shown). This also showed the tendency of the availability of keratinized mucosa around implants, and it may indicate that at least 2 mm of keratinized mucosa is preferable for the long-term success and survival of implants.

Our analysis showed that 16 of 223 implants were lost during the observation period. Among the six factors examined, only the implant position affected the cumulative implant survival rate and the main reason for implant failure was peri-implantitis (14/16 failed implants). However, the implant position did not affect the incidence of peri-implantitis. Compared to the mandible, the bone quality of the maxilla is lower [30] and the loading force is tilted to the implant axis. These factors might have acted as an exacerbating factor of peri-implantitis, resulting in the lower survival rate of the implants in the maxilla compared to the mandible.

Prosthetic complications occur due to the accumulation of mechanical damage to the implant, implant components, and supra-structures, resulting in the need for repairs and reconstructions of the implant prostheses, which may require time-consuming procedures and additional financial resources. The present investigation was a retrospective and multicenter study, and there were many differences in design patterns, materials, connections, and the attachment of supra-structures. It was therefore difficult to subdivide and review the factors that may affect the prosthetic survival rate, and only gender and type of prosthesis could be analyzed in this study.

The implant-supported fixed prostheses showed the highest complication-free survival rate in our study. It was reported that the veneering material’s chipping/fracture is the most common type of prosthetic complication for fixed prostheses [31, 32]. Pjetursson et al. reported that veneer fracture was observed in 13.5% of fixed prostheses after at least 5-year functioning [33]. In the present study, approx. 76% of the fixed prostheses were not veneered (metal occlusal surface), resulting in the lower complication rate after 25 years of functioning.

We also observed that the tooth-implant-supported prostheses had a lower complication-free rate than implant-supported fixed prostheses due to caries, periodontitis, or the root fracture of abutment teeth. Lang et al. reported that the survival rates of tooth implant-supported fixed partial dentures were 94.1% after 5 years and 77.8% after 10 years of functioning [31], and these results were almost the same as ours (93.9% after 5 years’ and 77.2% after 10 years’ functioning). Taking our results and those of Lang et al. into account, it appears that prosthetic complications of tooth implant-supported prostheses start arising after 7 years post-setting and then increase with time.

The implant-supported overdentures showed the lowest complication-free rate among the three implant types in the present study, due to the wear or fracture of artificial teeth, attachment fracture, and relines. Compared to another retrospective study of conventional complete dentures (without implant support) [34], our complication-free rate was higher and there was a difference in terms of the incidence of artificial tooth problems. That study showed a < 10% of incidence of artificial tooth problems during the first 5 years post-setting. The rigid support provided by an implant might have enhanced the loss, wear, and fracture of artificial teeth in our patients.

## Conclusions

In conclusion, our analyses revealed a cumulative survival rate of 89.8% of TPS-surface implants with at least 25 years of functioning. The survival rate of maxillary positioned implants was significantly lower than that of mandibulary positioned implants. The patient gender, implant location, and width of keratinized mucosa affected the rate of peri-implantitis, resulting in late failure. Implant-supported overdentures were frequently repaired compared to the fixed prostheses due to the wear or fracture of artificial teeth, attachment fracture, and relines. Tooth implant-supported prostheses were not beneficial for long term owing to the troubles of abutment tooth.
